# Supplementary material for: Alcam-a and Pdgfr-α are essential for the development of sclerotome-derived stromal cells that support hematopoiesis
Source: Nat Commun. 2023 Mar 1;14:1171. doi: 10.1038/s41467-023-36612-y (PMC9977867; doi:10.1038/s41467-023-36612-y)
Supplement: Supplementary file 1 — Supplementary information [file 41467_2023_36612_MOESM1_ESM.pdf]

Supplementary Information

Alcam-a and Pdgfr-α are essential for the development of sclerotome-derived stromal cells that support hematopoiesis

Murayama et al.

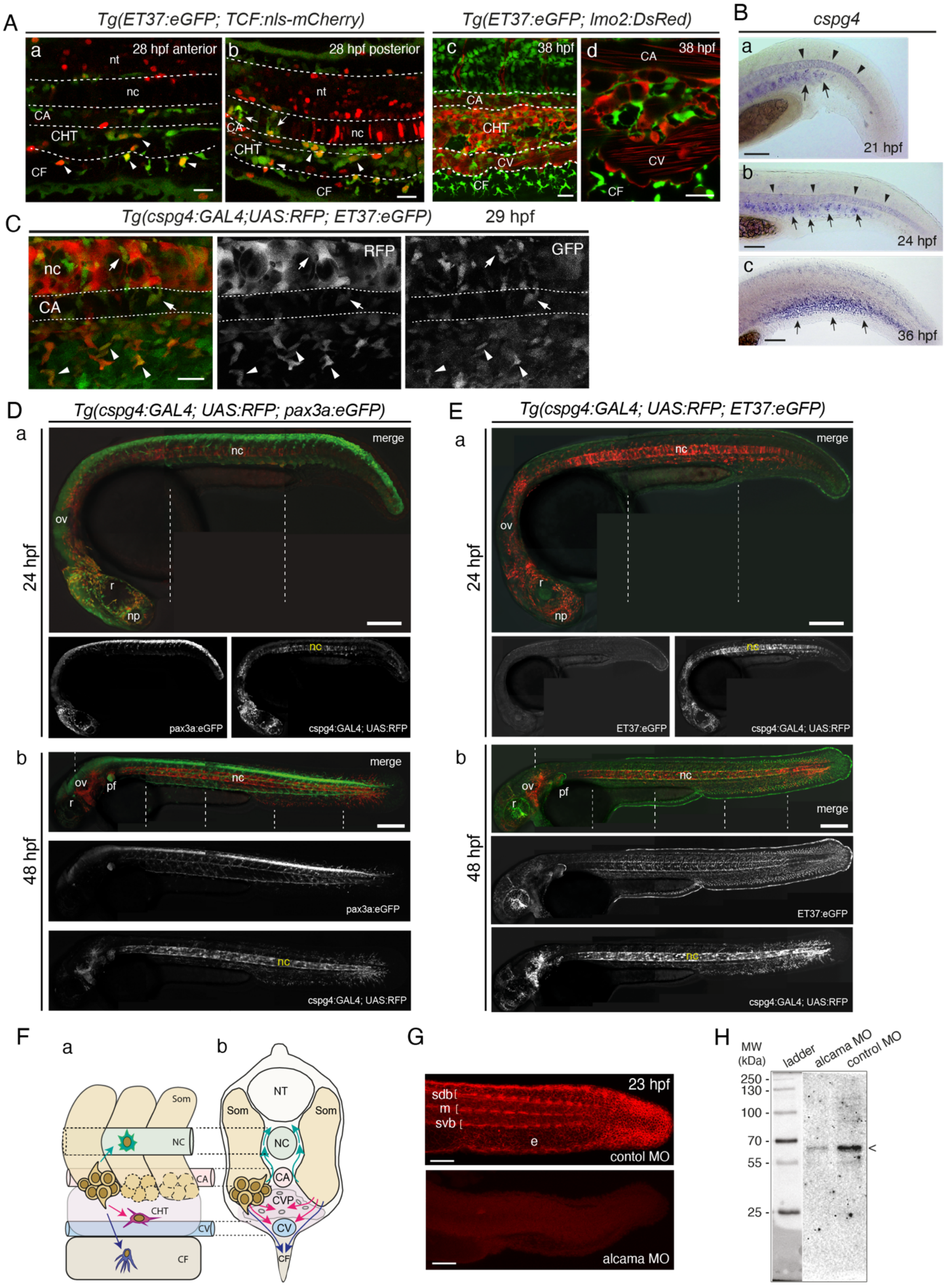

Supplementary Figure 1. Transgenic reporter lines used to study SCP development, and effects of alcama knockdown. (**A**) Confocal projections of the ventro-caudal region of *Tg(ET37:eGFP; TCF:nls-mCherry)* embryos at 28 hpf (**a**,**b**) and *Tg(ET37:eGFP; lmo2:DsRed)* embryos at 38 hpf (**c**,**d**), wherein DsRed highlights blood vessels and circulating primitive blood cells. Scale bars, 20 μm. (**B**)

WISH for *cspg4* at 21 (**a**), 24 (**b**) and 36 hpf (**c**). Arrows and arrowheads indicate somite VC derived cells and notochord, respectively. Scale bars, 100 µm. (**C**) Confocal projection of *Tg(ET37:eGFP; cspg4:GAL4; UAS:RFP)* embryo at 29 hpf. Dashed lines delineate the borders of the caudal artery. Scale bar, 20 µm. Arrows and arrowheads in (**A**) and (**C**) indicate dorsal- and ventral-wards migrating GFP^{+}/RFP^{+} VC derived cells, respectively. (**D,E**) Confocal projections of triple *Tg(cspg4:GAL4;UAS:RFP;pax3a:eGFP)* embryo (**D**) and *Tg(cspg4:GAL4;UAS:RFP; ET37:eGFP)* embryo (**E**) at 24 (**a**) and 48 (**b**) hpf. Dashed vertical lines show the joints of individual images. nc, notochord; np, nasal pit; ov, otic vesicle; pf, pectoral fin; r, retina. Scale bars, 200 µm. (**F**) Schematic representation of the caudal region in lateral (**a**) and transverse view (**b**), showing the migration paths of somite VC derived cells; magenta and blue arrows indicate the paths leading to CHT stromal cells and FMCs, respectively; green arrows show the dorsal-wards migration path (of putative chondrocyte and tenocyte progenitors). (**G**) Immunofluorescence for Alcama in control or alcama MO-injected embryo at 23 hpf. Representative images of n=20 for each condition from six independent experiments. Scale bars, 100 µm. (**H**) Immunoblot revealed by enhanced chemiluminescence showing the effect of the alcama MO on endogenous Alcama protein expression (second lane), in comparison to the uninjected control (third lane). 67.5 µg of total proteins were loaded per lane of a 12% acrylamide gel. Left: prestained MW markers (colorimetric image). Image from a single experiment. NT, neural tube; NC, notochord; CA, caudal artery; CVP, caudal venous plexus; CV, definitive caudal vein; CF, caudal fin; sdb, somite dorsal border; m, slow muscle pioneers prefiguring the horizontal myoseptum; svb, somite ventral border; e, epidermis.

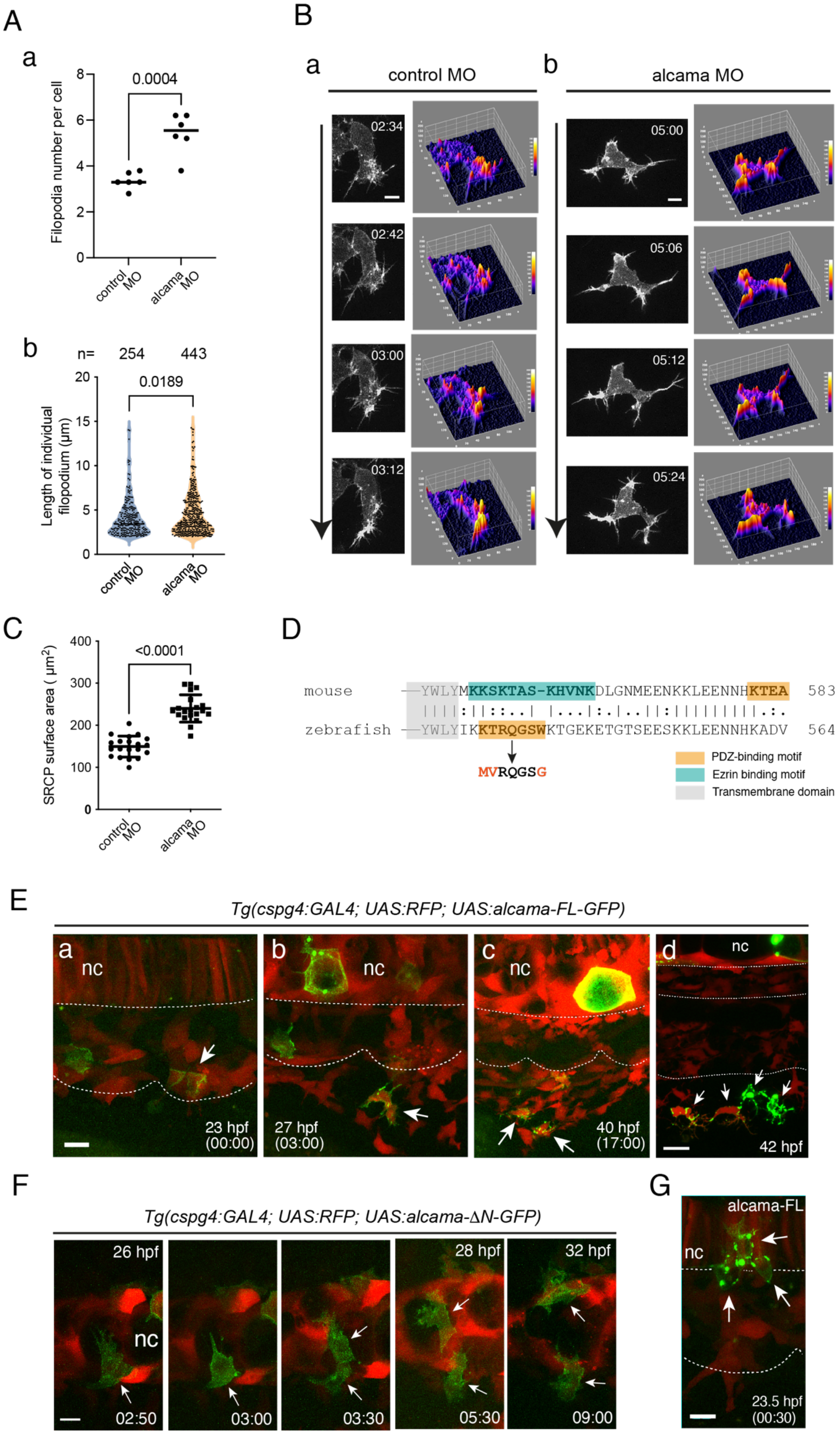

A
a
Filopodia number per cell
0.0004
control MO
alcama MO
b
n= 254 443
Length of individual filopodium (µm)
0.0189
control MO
alcama MO
B
a
control MO
02:34
02:42
03:00
03:12
b
alcama MO
05:00
05:06
05:12
05:24
C
SRCP surface area (µm²)
<0.0001
control MO
alcama MO
D
mouse —YWLYMKKKSKTAS-KHVNKDLGNMEENKKLEENNHKTEA 583
zebrafish —YWLYIKKTRQGSWKTGEKETGTSEESKKLEENNHKADV 564
MVRQGSG
PDZ-binding motif
Ezrin binding motif
Transmembrane domain
E
Tg(cspg4:GAL4; UAS:RFP; UAS:alcama-FL-GFP)
a nc 23 hpf (00:00)
b nc 27 hpf (03:00)
c nc 40 hpf (17:00)
d nc 42 hpf
F
Tg(cspg4:GAL4; UAS:RFP; UAS:alcama-ΔN-GFP)
26 hpf
nc
02:50
03:00
03:30
28 hpf
05:30
32 hpf
09:00
G
alcama-FL
nc
23.5 hpf (00:30)

Supplementary Figure 2. Alcama modulates SCP migration. (**A**) Quantification of filopodia number and length for migrating SCP leader cells in control and morphant embryos from the experiments analyzed in Fig. 2A-C (n=7 cells per condition, from 3 independent experiments). (**a**) Graph comparing the total number of filopodia ≥ 3 µm per cell during migration (averaged over 6 time-points; mean±SD; two-tailed Student's *t*-test). (**b**) Quantification of filopodia length (≥ 3 µm) for migrating SCP leader cells (measured at 7-10 time points; median±SD; two-tailed Mann-Whitney test.). (**B**) Surface plots of Lifeact-GFP intensity in the migrating SCPs shown in Fig. 2A; warmer colors represent higher intensity; vertical arrows, direction of migration. (**C**) Quantification of 2D surface area of SCPs in the *Tg(cspg4:GAL4; UAS:lifeact-eGFP)* embryos injected with control or alcama MO. n=20 cells for each, obtained in 8 embryos from three independent experiments. Mean±SD; two-tailed Student's *t*-test. (**D**) Alignment of amino acid sequences of the short cytoplasmic domain of mouse Alcam (CD166) and zebrafish Alcama. The light grey shading indicates the last residues of the transmembrane domain. Orange and green shadings indicate PDZ- or Ezrin-binding motif, and the PDZ-binding motif as mutated in this study is shown at the bottom. (**E**) Control cells expressing alcama-FL-eGFP construct showed a normal development, exemplified here by a GFP^+ cell followed over time from its appearance in a VC by 23 hpf (**a**, arrow), then as a ventral-wards emigrating SCP (**b**, arrow), that later underwent mitosis by 40 hpf (**c**, arrows); scale bar, 10 µm. (**d**) Alcama-FL-GFP^+ FMCs (arrows) at 42 hpf; scale bar, 20 µm. (**F**) Dorsal-wards migration of an Alcama-∆N-$eGFP^+$ VC cell (arrows) from 26 to 32 hpf; it underwent mitosis by 29.5 hpf, then one of the daughter cells migrated further dorsally. Scale bar, 10 µm. (**G**) Dorsal-wards migration of three Alcama-FL-GFP^+ VC cells (arrows) at 23.5 hpf. nc, notochord. Scale bar, 10 µm. Source data for **A** and **C** are provided as a Source Data file.

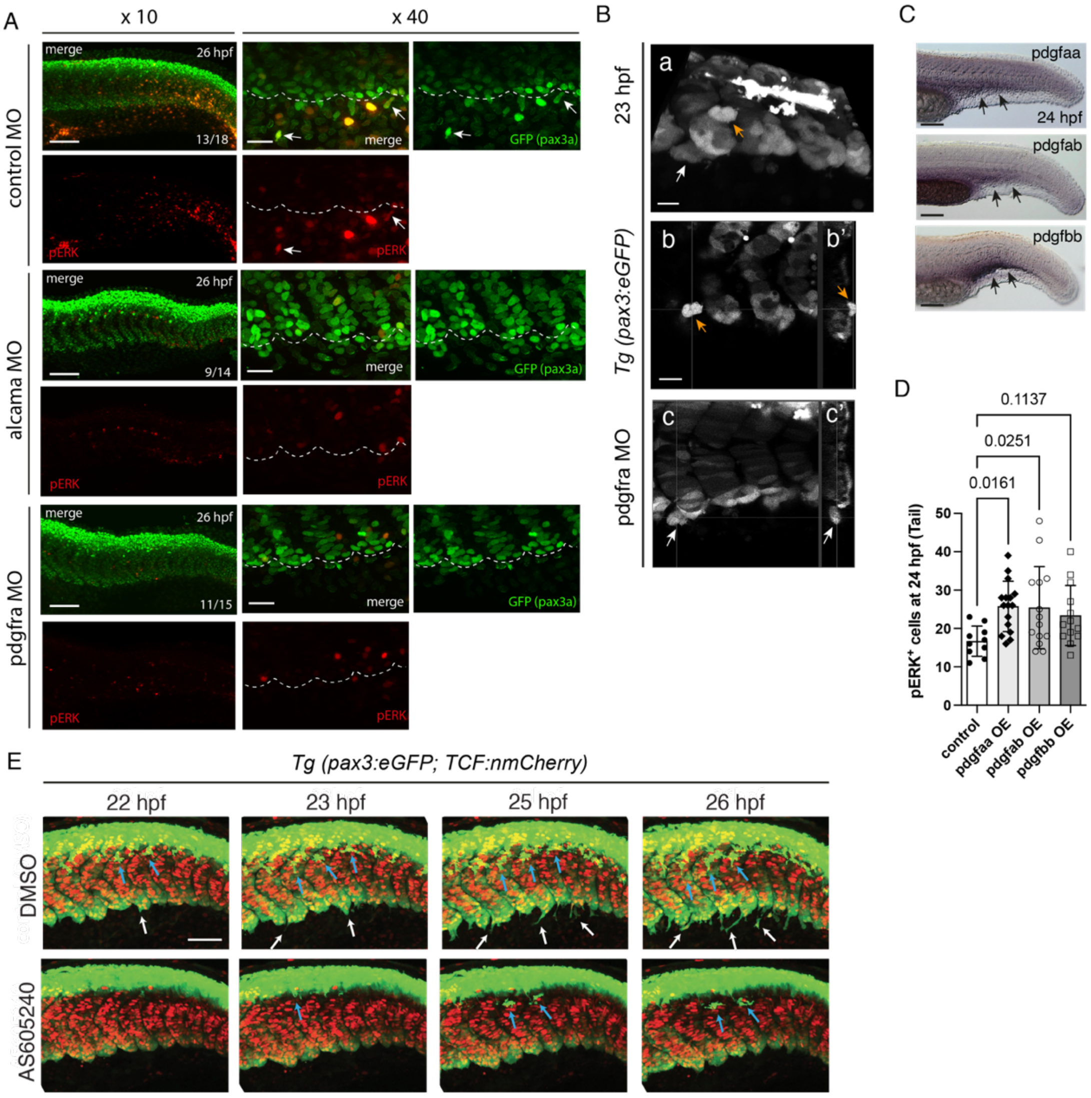

Supplementary Figure 3. Involvement of PDGFRα signaling in cluster cohesion and subsequent migration of SCPs. (**A**) Immunofluorescence at 26 hpf for pERK and GFP in *Tg(pax3a:eGFP)* embryos injected with control, alcama or pdgfra MO. Arrows point at pERK$^+$/GFP$^+$ SCPs and dashed lines delineate the ventral border of caudal somites. Representative images of n=13, 9 and 11 for control, alcama and pdgfra MO from 2 independent experiments. Scale bars, 100 µm (x10 panels) and 20 µm (x40 panels). (**B**) Cluster cohesion defects at 23 hpf in the *Tg(pax3a:eGFP)* pdgfra morphant embryo shown in Fig. 3B-b. White and orange arrows point at cell groups that appear to have detached from a VC laterally and medially. (**a**) Ventrally tilted maximum projection view; (**b, c)** single confocal planes; (**b', c')** optical transverse sections at the positions shown by a vertical line and arrow in **b** and **c**, respectively. Scale bars, 15 µm. (**C**) WISH for *pdgfaa*, *pdgfab* and *pdgfbb* at 24 hpf. Arrows point at signals at the ventral side of caudal somites. Scale bars, 100 µm. (**D**) Quantification of pERK$^+$ cells in the tail of control, pdgfaa-, pdgfab- and pdgfbb-overexpressing embryos at 24 hpf (n=10, 16, 14 and 13 embryos for control, pdgfaa, pdgfab and pdgfbb, respectively, from a single experiment. Mean±SD; One-way ANOVA followed by Dunnett's multiple comparison test). Source data are provided as a Source Data file. (**E**) Time-lapse confocal imaging from 22 to 26 hpf of *Tg(pax3a:eGFP; TCF:nls-mCherry)* embryos treated with 0.2 % DMSO (control) or 2 µM AS605240 from 20 hpf. White and blue arrows indicate migrating SCPs and neural crest cells, respectively. Note that in AS605240-treated embryos, cells expressing TCF:nmCherry (marking somitic origin) are missing in the dorsal-most region (dorsal to the somites), evidencing the absence of migration of dorsal somite cluster cells into the dorso-caudal fin. Scale bar, 100 µm.

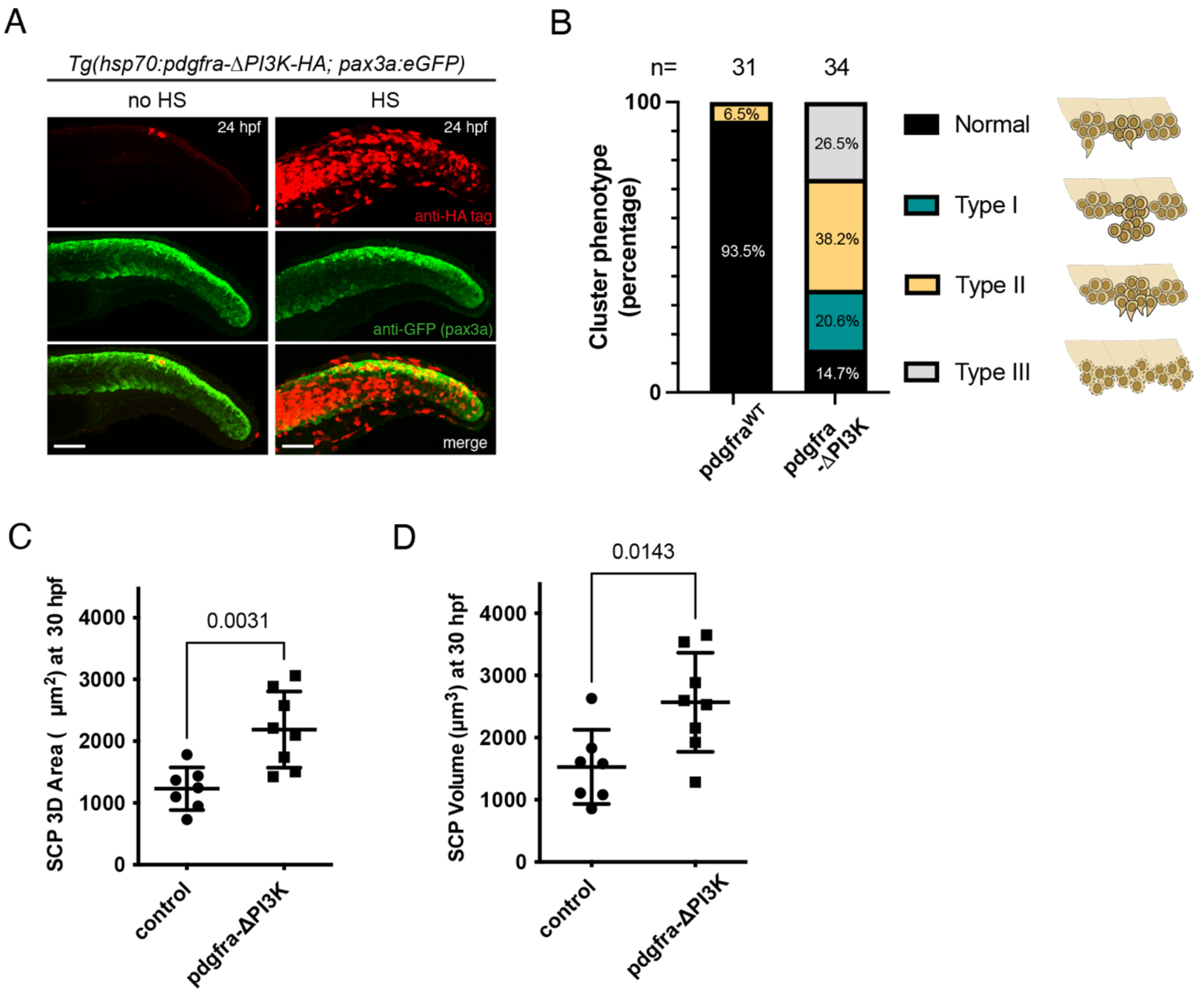

Supplementary Figure 4. Pdgfrα-ΔPI3K induces defects in cluster integrity, emergence and morphology of migrating SCPs. (**A**) Immunofluorescence at 24 hpf for HA-tag (red) and pax3a:eGFP (green) in the tail of hsp70:pdgfra-ΔPI3K-HA injected embryos without heat-shock (no HS) and after heat-shock at 20 hpf (HS). Representative images of n=6 for each condition from 2 independent experiments. Scale bars, 100 µm. (**B**) Bar graph representing the frequency of each VC phenotype in Pdgfra^{WT} and Pdgfra-ΔPI3K expressing embryos. Typical phenotypes observed at 23 hpf in hsp70:pdgfra-ΔPI3K heat-shocked embryos are shown; Type **I**, 'delamination/overflow' phenotype; Type **II**, 'simultaneous migration' phenotype; Type **III**, 'loose cluster' phenotype. n=31 and 34 for pdgfra^{WT} and pdgfra-ΔPI3K embryos, from 10 independent experiments. (**C**, **D)** 3D surface area (**C**) and volume (**D**) of individual SCP leader cells detached from their followers, for hsp70:pdgfra^{WT} and hsp70:pdgfra-ΔPI3K embryos at 30 hpf (n=7 and 8 cells for pdgfra^{WT} and pdgfra-ΔPI3K embryos, from two independent experiments; mean±SD; two-tailed Student's *t*-test). Source data for **B-D** are provided as a Source Data file

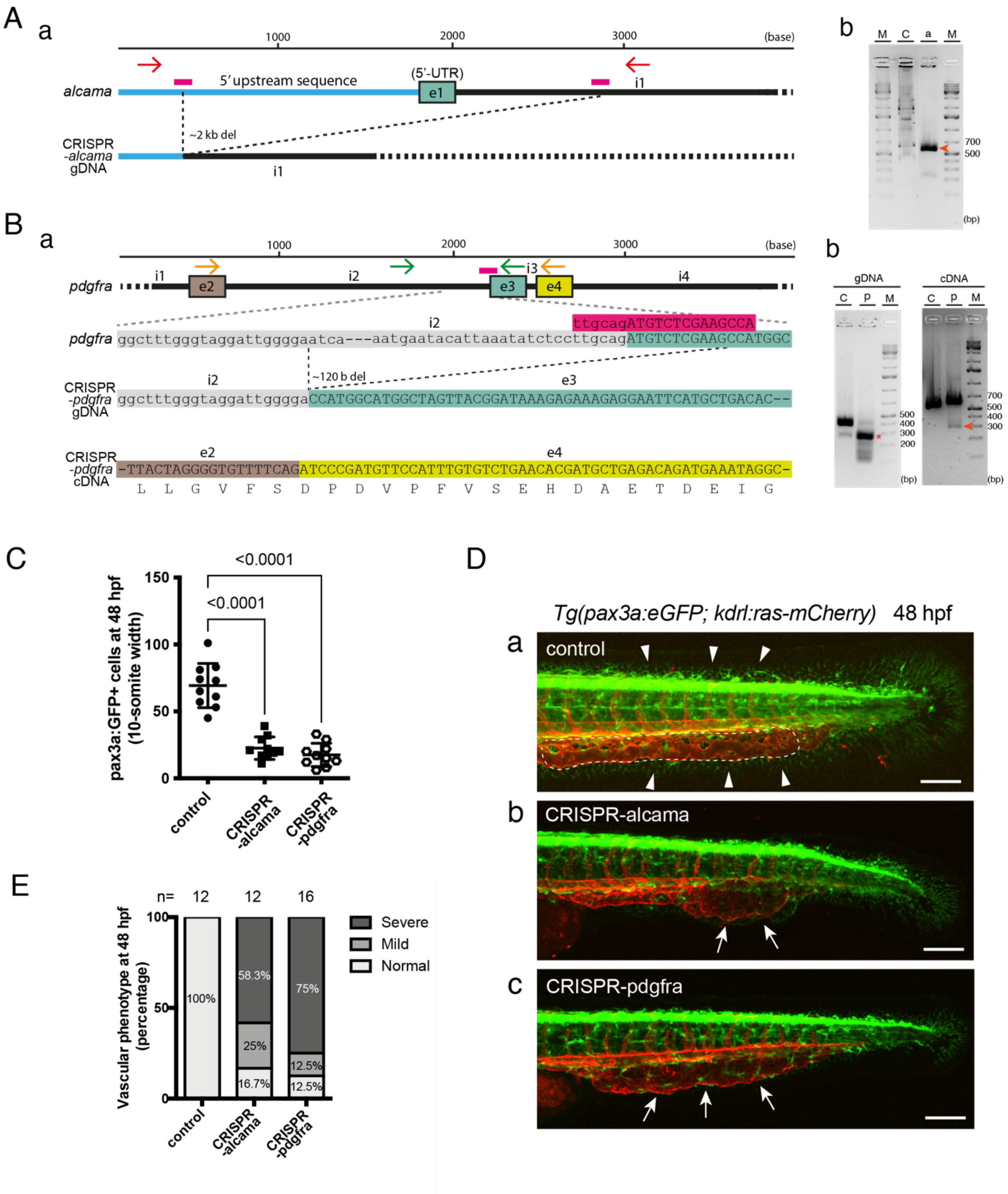

Supplementary Figure 5. Alcama- and pdgfra-crispant embryos phenocopy the alcama- and pdgfra-morphant phenotypes. (**A**) **a**, *alcama* gene with 5' upstream sequence indicated in blue followed by exon 1 and intron 1 in black. Guide RNAs (magenta) were designed to induce a deletion encompassing ~1.4 kb of promoter region, the non-coding exon1 and part of intron 1. **b**, Gel image of PCR products amplified with the primers shown in **a** (red arrows). using genomic DNA extracted from an individual embryo injected with the gRNAs (lane a), or an uninjected control embryo (lane C) at 48 hpf. Arrowhead points to the fragment specifically amplified in the alcama-Crispant, which was then sequenced after purification of the band from the gel. (**B**) **a**, Partial schematic structure of the *pdgfra* gene. Introns and exons are indicated by black lines and colored boxes, respectively. A single guide RNA (magenta) was designed at the intron 2 / exon3 junction, that led to a deletion of approximately

120 bp (CRISPR-pdgfra gDNA). **b**, Gel images of PCR products amplified with the primers shown in (**a)** using genomic (left) and complementary (right) DNAs as templates. Green and orange primer pairs in (**a)** were used for the amplification of genomic and complementary DNAs, respectively. gDNAs were extracted from individual embryos after live-imaging at 48 hpf. An asterisk indicates the fragment specifically amplified in the pdgfra-Crispant (lane p) and sequenced after purification of the band from the gel. cDNA was prepared from ~100 pdgfra-gRNA injected embryos (lane p) or uninjected control embryos (lane C) at 48 hpf. (**C**) Quantification of pax3a:eGFP$^+$ stromal cells in the CHT at 48 hpf. Counting was performed over a 10-somites width in control, alcama- and pdgfra-crispants (n=10 embryos for each group) from a single experiment (mean±SD; One-way ANOVA followed by Dunnett's multiple comparison test). (**D**) Confocal projections of uninjected control (**a**), alcama- (**b**) and pdgfra- (**c**) crispant *Tg(pax3a:eGFP; kdrl:ras-mCherry)* embryos at 48 hpf. In the control embryo (**a**), the dashed contour delineates the 10-somite wide area of the CHT wherein pax3a:eGFP$^+$ cells were counted in (**C)**, and arrowheads point to the mesenchymal cells that are missing in both the ventral and dorsal parts of the caudal fin in the crispant embryos. Arrows in (**b,c**) indicate CV plexus expansion to form a single tube in the crispant embryos. Scale bars, 200 µm. (**E**) Frequency histogram showing the incidence of abnormal CV plexus phenotype at 48 hpf. CV plexus morphology in each of the uninjected control (n=12), alcama (n=12) and pdgfra-crispant (n=16) *Tg(pax3a:eGFP; kdrl:ras-mCherry)* embryos was classified as 'Normal', 'Mild' or 'Severe' phenotype. 'Severe' and 'mild' phenotypes indicate a malformation in which the entire area or part of the caudal plexus was 'single-tubed', respectively. Source data for **C** and **E** are provided as a Source Data file.

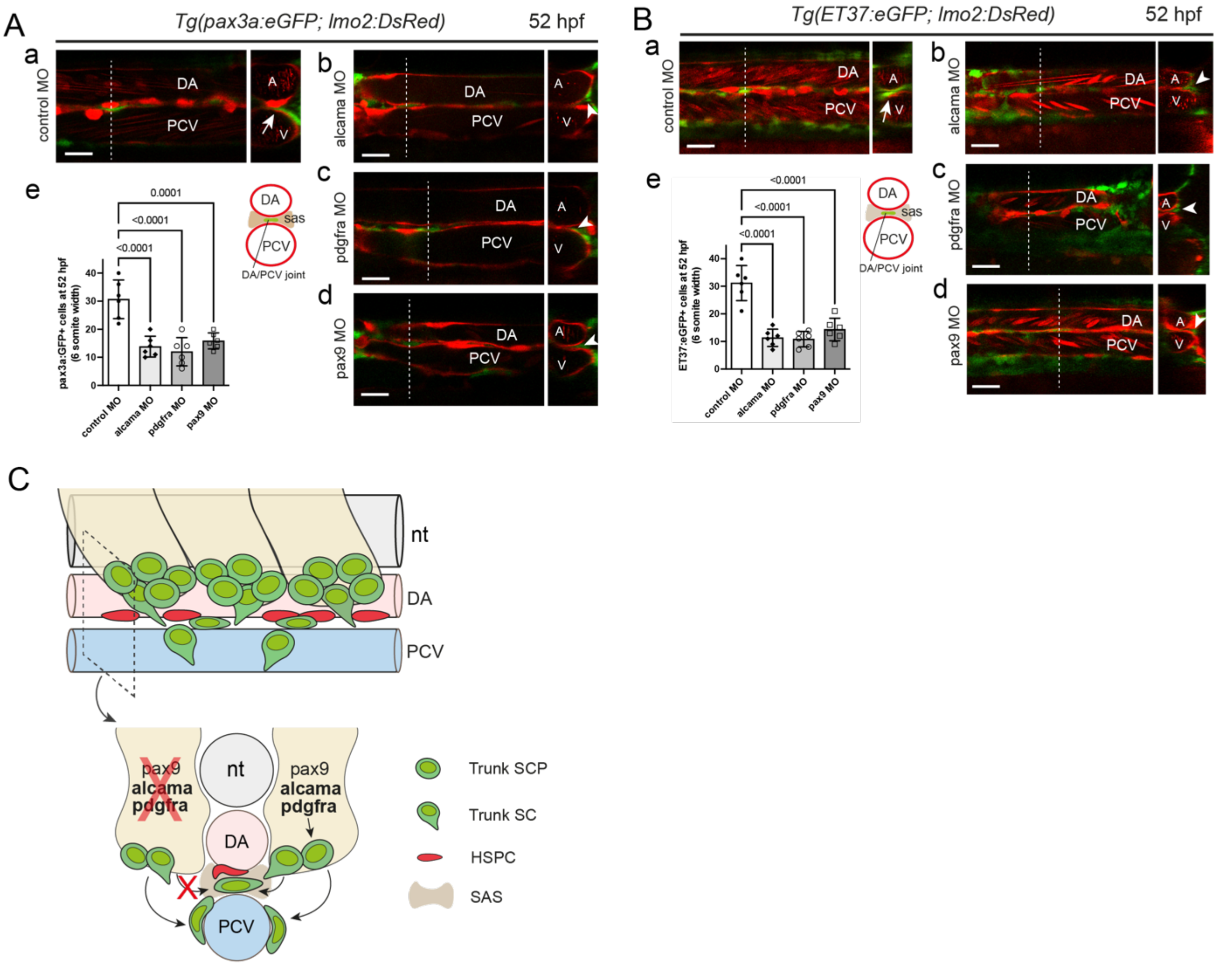

Supplementary Figure 6. Effect of Alcama, Pdgfr-α or Pax9 deficiency on stromal cell development in the trunk. A, B(a-d) Confocal sections at 52 hpf of the trunk region of *Tg(pax3a:eGFP; lmo2:DsRed)* embryos (**A**) or *Tg(ET37:eGFP; lmo2:DsRed)* embryos (**B**) injected with control (**a**), alcama (**b**), pdgfra (**c**) and pax9 (**d**) MOs. Dashed vertical lines indicate the position where a corresponding optical transverse section is shown to the right. Arrows and arrowheads point at stromal cells located in the DA/PCV joint or more lateral to it (sub-aortic space), respectively. Scale bars, 20 µm. **A, B-(e)**, Quantification of pax3a:eGFP^{+} (**A**) or ET37:eGFP^{+} (**B**) cells in live embryos injected with control, alcama, pdgfra or pax9 MO at 36 hpf. Counting was performed over a 6-somites width (n=6 embryos for each group; mean±SD; One-way ANOVA followed by Dunnett's multiple comparison test). DA or A, dorsal aorta; PCV or V, posterior cardinal vein; SAS, sub-aortic space. (**C**) A schematic diagram of stromal cell development in the trunk, showing that SCPs migrating from the somite VCs (sclerotome) position themselves just ventral to the DA, in contact with the hemogenic endothelium and HSPCs that emerge from it via EHT, and/or with the dorsal and lateral sides of the PCV, through which these HSPCs will enter circulation. The presence of these somite-derived stromal cells appears essential for HSPC emergence. Their close interaction with HSPCs in the SAS is disturbed in the absence of alcama, pdgfra, or pax9. Source data for **A-e** and **B-e** are provided as a Source Data file.

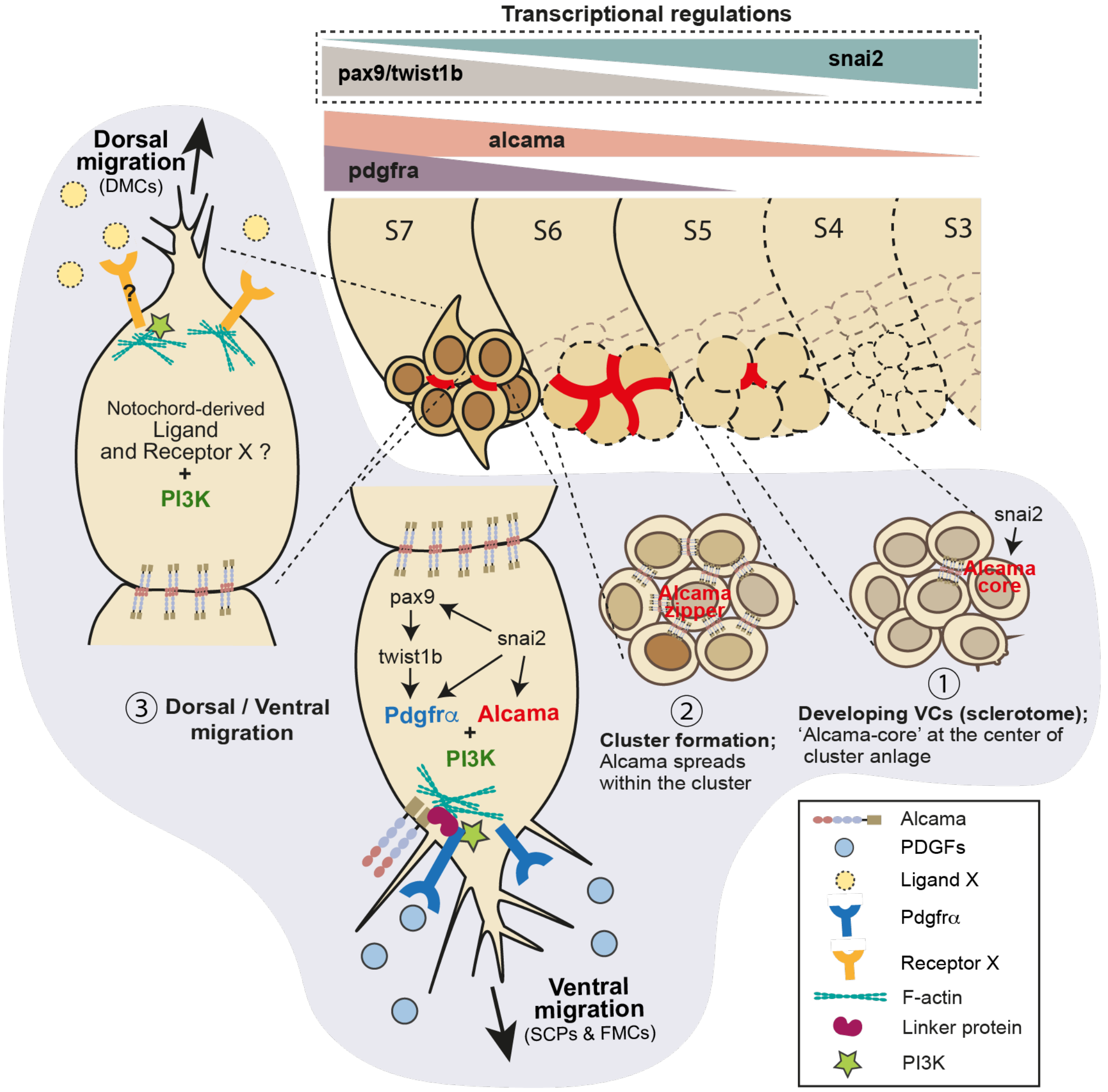

Supplementary Figure 7. Schematic model of SCP development. ① Sclerotome cells undergo an epithelial-to-mesenchymal transition (EMT) at the ventral part of caudal somites by maturation stage S5, making the sclerotome cluster morphologically apparent. Alcama first appears at the center of the cluster. ② Alcama progressively spreads to all cellular interfaces within the cluster (by S6), ensuring selective adhesion among them. ③ By stage S7, sclerotome cells initiate semi-collective dorsal- or ventral-ward migration to become dorsal mesenchymal cells or SCPs, respectively. Alcama and Pdgfr-α are involved in the regulation of F-actin in SCPs through a molecular crosstalk, and ventral migration is triggered by PI3K, which is activated downstream of Pdgfr-α stimulated by PDGFs. PI3K is also involved in the dorsal migration of sclerotome cells, but the upstream signals have not yet been identified. Among sclerotomal TFs, Snai2 activates *alcama* (likely indirectly) in younger somites, whereas Twist1b activates *pdgfra* expression in more developed somites. Since Pax9 shows inhibitory activity against *alcama* and *pdgfra* expression, and its expression level increases with somite maturation, it may exert its positive effect on sclerotome development by balancing the activating effects of Snai2 and Twist1b.

Supplementary tables

Supplementary Table 1. Primers used for BAC transgenesis

Primer	Sequence	Reference
pIndigobac_iTol2 F	TTCTCTGTTTTTGTCCGTGGAATGAACAATGGAAGTCC GAGCTCATCGCTCCCTGCTCGAGCCGGGCCCAAGTG	Suster et al.,2011 Bussmann and Schult-Merker, 2011
pIndigoBAC_iTol2 R2	AGCCCCGACACCCGCCAACACCCGCTGACGCGAACCC CTTGCGGCCGCATATTATGATCCTCTAGATCAGATC	Suster et al.,2011 Bussmann and Schult-Merker, 2011
Cspg4_GAL4FF F	CTCTCCAGGTCCCAAAGTGGCCACAGAGACTCAGAGA CTCGGACTAAAGTgccaccatgAAGCTACTGTCTTCTATCG AAC	This study
Cspg4_frt-kan R	aggtataggagtgccaggaagagggcagacaggagcggacacggggctct CCGCGTGTAGGCTGGAGCTGCTTC	This study

Supplementary Table 2a. Primers used for the synthesis of WISH probes

Primer	Sequence
alcama-WISH-F	CCTGCCGACGGTTATAGGTC
alcama-WISH-R	aagcttTAATACGACTCACTATAGGGGGCCGGTAATTCTTGGACCA
pdgfaa-WISH-F	TAGAAAGGCATGTTCCCCGG
pdgfaa-WISH-R	aagcttTAATACGACTCACTATAGGGAGAGTGATCCAAGAGCTGCG
pdgfbb-WISH-F	AAGAGCGGGGACAAAAGTGG
pdgfbb-WISH-R	aagcttTAATACGACTCACTATAGGGAAGAGCGGGGACAAAAGTGG
snai2-WISH-F	ACACTGAGAGGCCTGCATTC
snai2-WISH-R	aagcttTAATACGACTCACTATAGGGGGCATGTTCAAAACCTCAAACC

Supplementary Table 2b. Plasmids used for the synthesis of WISH probes

Gene name	Construct	Reference
cspg4	cspg4:pJC53.2	Wang et al., 2014[1]
myb	cmyb:pBK-CMV	Thompson et al., 1998[2]
pax9	pax9:pPICT3	Kudo et al., 2004[3]
pdgfab	pdgfab:pExpress1	This study
pdgfra	pdgfra:pCR4	Eberhart et al., 2008[4]
twist1a	twist1a:pExpress1	This study
twist1b	twist1b:pExpress1	This study

Supplementary Table 3. Antibodies used in this study

Antibody name	Dilution	Reference number, Manufacturer
zn-8 (anti-Alcama)	1:100	zn-8, DSHB
Phospho-p44/42 MAPK (ERK1/2)	1:100	#4370, Cell Signaling Technology
anti-HA.11 Epitope Tag	1:50	901501, Biolegend
Chicken anti-GFP	1:800	ab13970, Abcam
Rabbit anti-DsRed	1:300	632496, Takara
anti-Chicken-AlexaFluor 488	1:300	A-11039, ThermoFisher Scientific
anti-Mouse-HRP	1:300	F-21453, ThermoFisher Scientific
anti-Rabbit-HRP	1:300	G-21234, ThermoFisher Scientific
anti-Fluorescein-R-phycoerythrin	1:100	A21250, Invitrogen

Supplementary Table 4. Primers used for mutagenesis

UAS:alcama-ΔN-eGFP

Primer	Sequence
G-UAS-alcama-SP F	GACGCGTGGATCCACCGGTCGCCACGCCACCATGCATTCGGTTATCTGCCTTTTCGGTG
G-UAS-alcama-SP R	GTAGACTCACCTTCTCAGTGGGCGGCAGGCAGCTCCCTGG
G-UAS-alcama-dN F	TGCTCCAGGGAGCTGCCTGCCGCCCACTGAGAAGGTGAGTCTACAG
G-UAS-alcama R	TGAACAGCTCCTCGCCCTTGCTCACGCCTGCTCCGACATCTGCTTTATGATTGTTCTCCTCC

UAS:alcama-ΔPDZ-eGFP

Primer	Sequence
G-UAS-alcama-SP F	GACGCGTGGATCCACCGGTCGCCACGCCACCATGCATTCGGTTATCTGCCTTTTCGGTG
G-UAS-alcama-dPDZ R1	TCCGTCCGCCTGGCATTTCAGAGTCACATCATCAC
G-UAS-alcama-dPDZ F2	TGATGATGTGACTCTGAAATGCCAGGCGGACGGAAAC
G-UAS-alcama-dPDZ R2	TCC**C**GCTGCCTTGTCT**CACCA**TCTTGATATACAACCAGTAGATGAGTCCCACCAG
G-UAS-alcama-dPDZ F3	ATCAAGA**TGGTG**AGACAAGGCAGC**G**GGAAGACCGGAGAGAAGGAGAC
G-UAS-alcama R	TGAACAGCTCCTCGCCCTTGCTCACGCCTGCTCCGACATCTGCTTTATGATTGTTCTCCTCC

UAS:alcama-FL-eGFP

Primer	Sequence
G-UAS-alcama-SP F	GACGCGTGGATCCACCGGTCGCCACGCCACCATGCATTCGGTTATCTGCCTTTTCGGTG
G-UAS-alcama-dPDZ R1	TCCGTCCGCCTGGCATTTCAGAGTCACATCATCAC
G-UAS-alcama-dPDZ F2	TGATGATGTGACTCTGAAATGCCAGGCGGACGGAAAC
G-UAS-alcama R	TGAACAGCTCCTCGCCCTTGCTCACGCCTGCTCCGACATCTGCTTTATGATTGTTCTCCTCC

hsp70:pdgfra-ΔPI3K-HA

Primer	Sequence
G-hsp70-pdgfra-dn F1-2	CCTGGAATTCGGTACCCTCGAGGATATCGCCACCATGTTCCCGGTGCTGCCACAGTCAGTTCAGGCTC
G-hsp70-pdgfra-dn R1	AGGTTCAACACACCCTTGTAACTGGTCTCGCCGATTTTGAGGAGGTTGGTGCTG
G-hsp70-pdgfra-dn F2	GCACCAACCTCCTCAAAATCGGCGAGACCAGTTACAAGGGTGTGTTGAACCTG
G-hsp70-pdgfra-dn R2	TGGGGACG**A**ACTGCATGGTGTCTGCCTGCTTCATGTCCATA**A**AATCACCTTTCCCCTC
G-hsp70-pdgfra-dn F3	AGGTGATTT**T**TATGGACATGAAGCAGGCAGACACCATGCAGTT**T**CGTCCCCATGCTGGAAATG
G-hsp70-pdgfra-dn R3 HA-tag	GTATGGCTGATTATGATCGCGGCCGCGGATCCTCATGCGTAATCAGGCACATCATAAGGATAAGCGTAGTCTGGGACGTCGTATGGGTAGCCTGCTCCCAGGAAGCTGTCCTCCACCAGGTC

hsp70:pdgfra^{WT}-HA

Primer	Sequence
G-hsp70-pdgfra-dn F1-2	CCTGGAATTCGGTACCCTCGAGGATATCGCCACCATGTTCCCGGT GCTGCCACAGTCAGTTCAGGCTC
G-hsp70-pdgfra-dn R1	AGGTTCAACACACCCTTGTAACTGGTCTCGCCGATTTTGAGGAGGT TGGTGCTG
G-hsp70-pdgfra-dn F2	GCACCAACCTCCTCAAAATCGGCGAGACCAGTTACAAGGGTGTGTT GAACCTG
G-pdgfra-wt R1	TGGGGACGTACTGCATGGTGTCTGCCTGCTTCATGTCCATATAATC AC
G-pdgfra-wt F1	AGGTGATTATATGGACATGAAGCAGGCAGACACCATGCAGTACGTC
G-hsp70-pdgfra-dn R3 HA-tag	GTATGGCTGATTATGATCGCGGCCGCGGATCCTCATGCGTAATCAG GCACATCATAAGGATAAGCGTAGTCTGGGACGTCGTATGGGTAGC CTGCTCCCAGGAAGCTGTCCTCCACCAGGTC

Supplementary Table 5. Primers used for cloning

Primer	Sequence
Alcama_SC_frg1 F1	GCCGGACTGTATAAAGGAGAACC
Alcama_SC_frg1 R1	AAGCTCACTGTGACGATCTGAG
Alcama_SC_frg2 F2	AAGGGAAAGAAGGTCACGGTG
Alcama_SC_frg2 R2	AGTGTGACAAGGCCTCTTTCC
Pdgfaa_SC F1	CTGCGCTGGGACACTTTTG
Pdgfaa_SC R1	AGAGTGATCCAAGAGCTGCG
Pdgfab_SC F1	GGTGCATCGGGTCATTTATAG
Pdgfab_SC R1	ATGTGGTTTCACCTCATGTCC
Pdgfbb_SC F1	ATATTTGCTCGCGTTAAGTGG
Pdgfbb_SC R1	GATTGCATCCCTCTGAACATC
Twist1b_SC F1	ACCCTCATGCTGGAATAACG
Twist1b_SC R1	TCCTCGTGTTTTCCCAGCTC
G-pax9-ORF5' F	ACACTATAGAACAAGTTTCGGTCCGGAATTCGTTACCATGGAGCCAGC CTTTGGGGAG
G-pax9-ORF3' R	TACGACTCACTATAGGGACCACTCCTCGAGTCACAGCTGTGGGGAGA GAGAGC
G-snai2-ORF5’ F	ACTATAGAACAAGTTTCGGTCCGGAATTCGTTACCATGCCTCGTTCATT CCTAGTAAAG
G-snai2-ORF3’ R	ATACGACTCACTATAGGGACCACTCCTCGAGTCAGTGTGCGATGCAAC AGCCAG

Supplementary Table 6. MOs used in this study

MO name	MO sequence (5' -> 3')	reference
MO1-alcama	GTCCGGCGACAGTCTCAATAGAGAG	Choe et al., 2013
MO1-pax9	CCAAAGGCTGGCTCTAGTTATGCAG	Swartz et al., 2011<br>Charbord et al., 2014
MO2-pdgfra	TTCGAGACATCTGCAAGGAGATATT	French et al., 2014
MO1-snai2	ATACATGTCATTTTCTCACCCGTGT	Charbord et al., 2014<br>Bickers et al., 2018
MO1-twist1a	ACCTCTGGAAAAGCTCAGATTGCGG	Das et al., 2012
MO3-twist1b	TTAAGTCTCTGCTGAAAGCGCGTG	Das et al., 2012

Supplementary Table 7. Primers used for qPCR

Primers	Sequence
alcama-qPCR-F	AGTGGAGTGTCAACGGAACC
alcama-qPCR-R	CGAGTTTATTGGTCACGAGGC
pax9-qPCR-F	CCACTCTTCCTGGACATATGGC
pax9-qPCR-R	TTCTAGTTTGGCGCTGGGAAAG
pdgfra-qPCR-F	ACGCTGAGTGATGTCTGGTC
pdgfra-qPCR-R	AGGTTTGGTCATTCGGTATCC
snai2-qPCR-F	TGCAGGGACACATTAGAACAC
snai2-qPCR-R	TGCACTGGTATTTCTTCACGTC
twist1a-qPCR-F	TGTCAACATCCCACTAACGCAC
twist1a-qPCR-R	TGACGCTCCAGAATTTTCCC
twist1b-qPCR-F	ACACAAAGTTGCTTGGAATACC
twist1b-qPCR-R	CTCGCTTAAGTCTCTGCTG

Supplementary Table 8. Primers used for the cloning of alcama and pdgfra promoters

Primer	Sequence
G alcama promoter F1-2	TCTTACGCGTGCTAGCCCGGGCTCGAGCAGCATTGATGGTTCCATGAAGA
G alcama promoter R1-2	GAAACCAACGTAGGACCTTAAGACCCTCCAGGTAAGAACATG
G alcama promoter F2	GACATGTTCTTACCTGGAGGGTCTTAAGGTCCTACGTTGGTTTC
G alcama promoter R2-2	GCTGCAACCTATCACTGGACAACATCCATACACTTTAACACAAAATATTTAAC
G alcama promoter F3-2	GTGTTAAAGTGTATGGATGTTGTCCAGTGATAGGTTGCAGC
G alcama promoter R3-2	CTTTACCAACAGTACCGGAATGCCAAGCTTATTGAGAGTTGAGTGCGCTG
G pdgfra promoter F1	TTACGCGTGCTAGCCCGGGCTCGAGTCACAATATATCTGTTTCATAAGG
G pdgfra promoter R1	CATCGTCTAACCACTCTTATAAAGTCGTTAACAACATTATAAGTAGC
G pdgfra promoter F2	CAAGCTACTTATAATGTTGTTAACGACTTTATAAGAGTGGTTAGAC
G pdgfra promoter R2	CATGGCAGGCTGCAGATAATGGCTCATTATGAATTAGTACCCTACG
G pdgfra promoter F3	GTCGCGTAGGGTACTAATTCATAATGAGCCATTATCTGCAGCCTG
G pdgfra promoter R3	CTTTACCAACAGTACCGGAATGCCAAGCTTAGCTTAATGCTACAGTCCATC

Supplementary Table 9. Injection conditions for Luciferase assay (amount per egg)

pGL-alcama-luc	pRL-TK	pax9 MO	pax9 mRNA	snai2 MO	snai2 mRNA
15 pg	1.5 pg	6 ng	-	-	-
		-	50 pg	-	-
		-	-	8 ng	-
		-	-	-	100 pg

pGL-pdgfra-luc	pRL-TK	snai2 MO	snai2 mRNA	twist1a+1b MO	twist1b mRNA
15 pg	1.5 pg	8 ng	-	-	-
		-	100 pg	-	-
		-	-	2 ng each	-
		-	-	-	100 pg

Supplementary Table 10. gRNAs and primers used for CRISPR experiment

gRNA	Sequence 5' → 3'
alcama-intron1	CTGTCTGCCTAATGACAAAG
alcama-intron2	GGAGTCCGTGATCGGGGGAC
pdgfra-i2e3	TTGCAGATGTCTCGAAGCCA

Primer	Sequence 5' → 3'
alcama-i1-F	TGTAAATGTGCTGCTTGTGG
alcama-i1-R	TAATGAGAGTAGGCTGTGCA
pdgfra-i2-F	GGCTTTGGGTAGGATTGGGG
pdgfra-e3-R	GCTGGCAGGTGTAGAAACGA
pdgfra-e2-F	TGGGATTGTCTTACTAGGGGTG
pdgfra-e4-R	ATTGTCCTGGCGGTATTGTAC

Uncropped scans ; red squares indicate the area used for each figures.

Fig S1-H

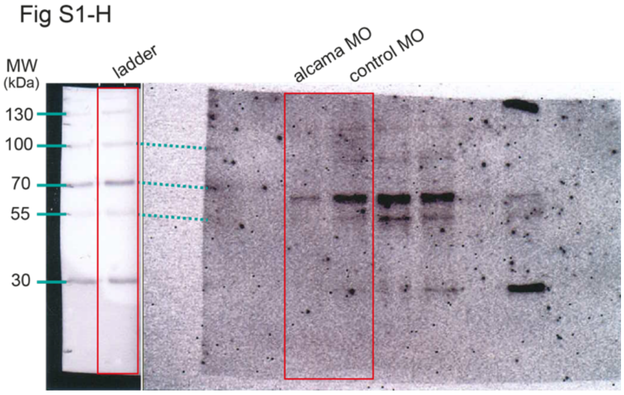

Fig S5, A-b

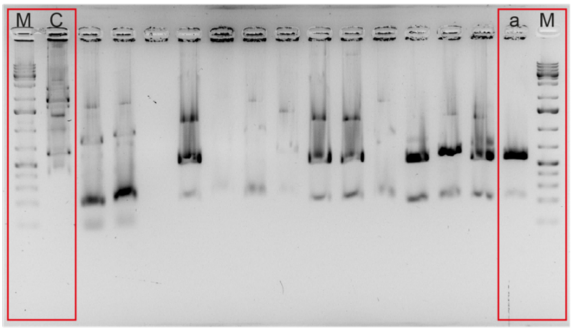

Fig S5, B-b, left

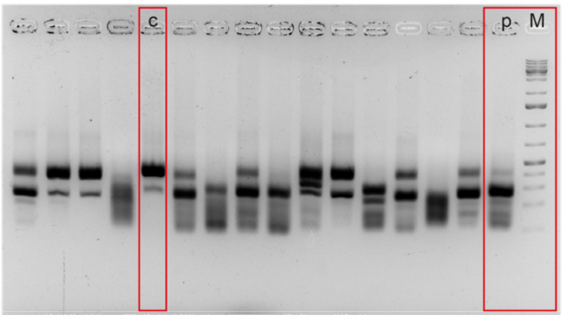

Fig S5, B-b, right

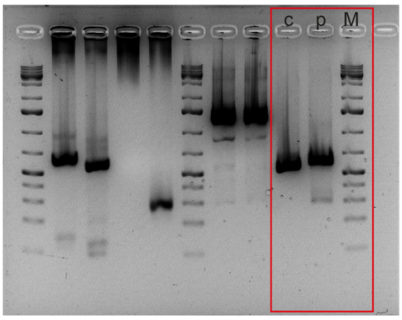

Supplementary references

1. Wang, Y. Y., Pan, L. Y., Moens, C. B. & Appel, B. Notch3 establishes brain vascular integrity by regulating pericyte number. *Dev.* **141**, 307–317 (2014).
2. Thompson, M. A. *et al.* The cloche and spadetail genes differentially affect hematopoiesis and vasculogenesis. *Dev. Biol.* **197**, 248–269 (1998).
3. Kudo, H., Amizuka, N., Araki, K., Inohaya, K. & Kudo, A. Zebrafish periostin is required for the adhesion of muscle fiber bundles to the myoseptum and for the differentiation of muscle fibers. *Dev. Biol.* **267**, 473–487 (2004).
4. Eberhart, J. K. *et al.* MicroRNA Mirn140 modulates Pdgf signaling during palatogenesis. *Nat. Genet.* **40**, 290–298 (2008).
