# Supplementary material for: Alcam-a and Pdgfr-α are essential for the development of sclerotome-derived stromal cells that support hematopoiesis
Source: Nat Commun. 2023 Mar 1;14:1171. doi: 10.1038/s41467-023-36612-y (PMC9977867; doi:10.1038/s41467-023-36612-y)
Supplement: Supplementary file 3 — Description of Additional Supplementary Files [file 41467_2023_36612_MOESM3_ESM.pdf]

Description of Additional Supplementary Files

File name: **Supplementary Movie 1**
Description: **VE-DIC/Nomarski imaging of ventral somite cells forming clusters.** The caudal region covering somite maturation stages S4 to S6 in a wild-type embryo was imaged every 10 sec. for 48 min. Arrows indicate the central lumen observed upon cluster formation. Scale bar, 20 µm.

File name: **Supplementary Movie 2**
Description: **pax3a:eGFP^{medium} expression marks somite VCs and SCPs emigrating from them.** 3D rendering from confocal imaging of a live *Tg(pax3a:eGFP; TCF:nls-mCherry)* embryo, followed by time-lapse imaging every 6 min for 8 hrs, starting at 24 hpf. Anterior to the left, dorsal to the top. pax3a:eGFP^{medium} stromal progenitors emigrate in ventral direction from the ventral border of caudal somites. Some of these cells have inherited TCF:nls-mCherry^{+} from their somitic origin. Pax3a:eGFP^{high} neural crest cell derived pigment cell (PC) precursors are also seen migrating, from the dorsal side of the spine over the medial side of somites and then beginning to arrive in the CHT by the end of the sequence. NC, notochord; NT, neural tube; SVC, somite ventral cluster.

File name: **Supplementary Movie 3**
Description**: Live imaging of an Alcama-FL-eGFP^{+} SCP.** Following injection of a *Tg(cspg4:Gal4;UAS:RFP)* embryo with a UAS:alcama-FL-eGFP construct at the 1-cell stage, GFP^{+} SCPs were imaged for 20 hours every 6 min. from 23 hpf. The left panel shows the overlay of RFP and GFP signals, and the intense GFP signal at cell contact points. The main GFP^{+} cell followed up here undergoes mitosis at t=10h36, after which (by t=11h) intense GFP signals underline the interface of the two daughter cells. Scale bar, 20 µm.

File name: **Supplementary Movie 4**
Description: **Live imaging of an Alcama-ΔN-eGFP^{+} SCP.** Following injection of a *Tg(cspg4:Gal4;UAS:RFP)* embryo with a UAS:alcama-ΔN-eGFP construct at the 1-cell stage, a GFP^{+} SCP was imaged for 16 hours every 6 min. from 23 hpf. The left panel shows the overlay of RFP and GFP signals, and the right panel the GFP signal only. Scale bar, 10 µm.

File name: **Supplementary Movie 5**
Description: **Live imaging of an Alcama-ΔPDZ-eGFP^{+} SCP.** Following injection of a *Tg(cspg4:Gal4;UAS:RFP)* embryo with a UAS:alcama-ΔPDZ-eGFP construct, a GFP^{+} SCP was tracked for 7 hours. Images were acquired at 6 min intervals from 25 hpf. The left panel shows the overlay of RFP and GFP signals, and the right panel the GFP signal only. Scale bar, 10 µm.

File name: **Supplementary Movie 6**
Description: **Pdgfra deficiency affects the number and migration range of SCP-derived stromal cells, and thereby CV plexus structure.** Time-lapse confocal imaging of *Tg(pax3a:eGFP; lmo2:DsRed)* embryos injected with control or pdgfra MO. Images were acquired from 38 hpf for 20 hrs at 6 min intervals. GFP^{high} cells are mostly neural crest-derived, including pigment cells migrating into the CHT. Somite VC-derived stromal cells and FMCs are GFP^{medium}. The Lmo2:Dsred transgene highlights vascular cells and more weakly circulating blood cells. In the morphant embryo, the endothelial cells that formed the venous plexus did not migrate further ventral-wards than the VC-

derived stromal cells, and this led to a correspondingly narrower venous plexus, often reduced to a single convoluted tube. Anterior to the left. Scale bar, 30 µm.

File name: **Supplementary Movie 7**
Description: **Pdgfra-ΔPI3K expressing SCPs show abnormal migration behavior and morphology.** Time-lapse confocal imaging of Lifeact-GFP+ SCPs in *Tg(cspg4:GAL4; UAS:lifeact-GFP)* embryos ubiquitously expressing $pdgfra^{WT}$ or pdgfra-ΔPI3K from 20 hpf. Images were acquired for 10 hrs at 6 min intervals from 24 hpf. GFP^{+} SCPs were located in both cases at the ventral border of a somite VC at timepoint zero. Scale bar, 10 µm.

File name: **Supplementary Movie 8**
Description: **Spatial localization of pax3a:$eGFP^{+}$ cells around the developing dorsal aorta.** Confocal z-stack acquisition of a live *Tg(pax3a:eGFP; kdrl:ras-mCherry)* embryo at 26 hpf, wherein farnesylated mCherry delineates the contour of endothelial cells. In the right panel, the bright-field image is overlaid on the fluorescence images in order to show the anatomical structures. DA, dorsal aorta; PCV, posterior cardinal vein. Scale bar, 25 µm.

File name: **Supplementary Movie 9**
Description: **Dynamics of pax3a:$eGFP^{+}$ mesenchymal cells in the sub-aortic space.**
Time-lapse confocal imaging of a *Tg(pax3a:eGFP; kdrl:ras-mCherry)* embryo. Images were acquired for 17 hrs at 6 min. intervals from 42 hpf. Scale bar, 50 µm.
