# Supplementary material for: Alcam-a and Pdgfr-α are essential for the development of sclerotome-derived stromal cells that support hematopoiesis
Source: Nat Commun. 2023 Mar 1;14:1171. doi: 10.1038/s41467-023-36612-y (PMC9977867; doi:10.1038/s41467-023-36612-y)
Supplement: Supplementary file 13 — Reporting Summary [file 41467_2023_36612_MOESM13_ESM.pdf]

nature portfolio

Corresponding author(s): Emi Murayama

Last updated by author(s): 2023/01/25

Reporting Summary

Nature Portfolio wishes to improve the reproducibility of the work that we publish. This form provides structure for consistency and transparency in reporting. For further information on Nature Portfolio policies, see our Editorial Policies and the Editorial Policy Checklist.

Statistics

For all statistical analyses, confirm that the following items are present in the figure legend, table legend, main text, or Methods section.

n/a	Confirmed	
☐	☒	The exact sample size (*n*) for each experimental group/condition, given as a discrete number and unit of measurement
☐	☒	A statement on whether measurements were taken from distinct samples or whether the same sample was measured repeatedly
☐	☒	The statistical test(s) used AND whether they are one- or two-sided<br>*Only common tests should be described solely by name; describe more complex techniques in the Methods section.*
☒	☐	A description of all covariates tested
☐	☒	A description of any assumptions or corrections, such as tests of normality and adjustment for multiple comparisons
☐	☒	A full description of the statistical parameters including central tendency (e.g. means) or other basic estimates (e.g. regression coefficient) AND variation (e.g. standard deviation) or associated estimates of uncertainty (e.g. confidence intervals)
☐	☒	For null hypothesis testing, the test statistic (e.g. *F*, *t*, *r*) with confidence intervals, effect sizes, degrees of freedom and *P* value noted<br>*Give P values as exact values whenever suitable.*
☒	☐	For Bayesian analysis, information on the choice of priors and Markov chain Monte Carlo settings
☒	☐	For hierarchical and complex designs, identification of the appropriate level for tests and full reporting of outcomes
☒	☐	Estimates of effect sizes (e.g. Cohen's *d*, Pearson's *r*), indicating how they were calculated

Our web collection on statistics for biologists contains articles on many of the points above.

Software and code

Policy information about availability of computer code

Data collection	The following softwares were used for image aquisition: LAS X 3.5.7.23225 (Leica), MetaMorph 7.10.1.161 (Molecular Devices), Image Lab 6.0 for GelDoc (Bio-Rad), Image Lab 6.1 for Chemidoc MP (BioRad) and GloMax Discover System sostware 4.0.0 (Promega) and Python 3.8.6.
Data analysis	Fiji (version 2.9.0) and IMARIS (version 6.8.1) were used for image analysis and quantification. Snapgene viewer (version 6.2) was used for sequence analysis. GraphPad Prism 9 (version 9.4.1) was used for statistical analysis. Adobe Photoshop (version 23.5.1) and Illustrator (version 26.5) were used for figure preparations.

For manuscripts utilizing custom algorithms or software that are central to the research but not yet described in published literature, software must be made available to editors and reviewers. We strongly encourage code deposition in a community repository (e.g. GitHub). See the Nature Portfolio guidelines for submitting code & software for further information.

Data

Policy information about availability of data

All manuscripts must include a data availability statement. This statement should provide the following information, where applicable:

- Accession codes, unique identifiers, or web links for publicly available datasets
- A description of any restrictions on data availability
- For clinical datasets or third party data, please ensure that the statement adheres to our policy

All data are available in the manuscript or supplemental materials. Raw imaging files are available upon request due to their large size. Source data are provided

with this paper.
Following databases are used in this study;
ZFIN databank (https://zfin.org) for in situ hybridization data
Ensembl genome blowser, GRCz11 (https://www.ensembl.org/Danio_rerio/Info/Index) for genomic / cDNA analysis

Human research participants

Policy information about studies involving human research participants and Sex and Gender in Research.

Reporting on sex and gender	Not relevant to our work.
Population characteristics	Not relevant to our work.
Recruitment	Not relevant to our work.
Ethics oversight	Not relevant to our work.

Note that full information on the approval of the study protocol must also be provided in the manuscript.

Field-specific reporting

Please select the one below that is the best fit for your research. If you are not sure, read the appropriate sections before making your selection.

☒ Life sciences ☐ Behavioural & social sciences ☐ Ecological, evolutionary & environmental sciences

For a reference copy of the document with all sections, see nature.com/documents/nr-reporting-summary-flat.pdf

Life sciences study design

All studies must disclose on these points even when the disclosure is negative.

Sample size	Sample size was determined based on consensus practice in the field.<br>Basically, for confocal in vivo images showing representative results, a total of n= 6 or more embryos were analyzed with consistent results.<br>Since timelapse imaging is limited to processing 3-4 embryos at a time, the number of experiments was increased in such cases.<br>For the cell tracking, 17 or more cells in 5 or more individuals were analyzed from 4 independent experiments.<br>For the photoconversion experiment, we analyzed a total of 42 cells in 10-12 embryos from three independent runs.<br>For luciferase assay, a biological triplicate was set up and basically three or more independent experiments were performed with at least n=6 (pooled tail lysate extracted from 10-15 tails).<br>For qPCR, a biological triplicate was set up and basically three or more independent experiments were performed with at least n=3 (pooled cDNA extracted from 40 tails).<br>For WISH and immunofluorescense, 6-10 embryos were analyzed for each time with 3 or more independent experiments.
Data exclusions	No data were excluded from analysis.
Replication	Biological replicates were conductd and they reproduced all findings. Number of replicates in indicated in the Figure legends.
Randomization	In each experiment, samples were selected based on viability and normal development using a population of samples from the same parents as far as possible. In addition, transgenic embryos are further sorted to select samples that express the desired fluorescent protein. From this population of embryos, samples are randomly selected.<br>All samples used for immunostaining or in situ hybridization were randomly selected from each treatment group.
Blinding	The authors were blinded to group allocation during data collection but not during data analysis because the phenotypes were quite distinct between the control and testing embryos.For CRISPR experiment, gRNA-injected F0 embryos were genotyped after phenorypical analysis.<br>Blinding was not performed for MO-injected embryos.

Reporting for specific materials, systems and methods

We require information from authors about some types of materials, experimental systems and methods used in many studies. Here, indicate whether each material, system or method listed is relevant to your study. If you are not sure if a list item applies to your research, read the appropriate section before selecting a response.

Materials & experimental systems

n/a	Involved in the study
☐	☒ Antibodies
☒	☐ Eukaryotic cell lines
☒	☐ Palaeontology and archaeology
☐	☒ Animals and other organisms
☒	☐ Clinical data
☒	☐ Dual use research of concern

Methods

n/a	Involved in the study
☒	☐ ChIP-seq
☒	☐ Flow cytometry
☒	☐ MRI-based neuroimaging

Antibodies

Antibodies used	Following antibodies were used in this study;<br>zn-8 (anti-Alcama) 1:100 zn-8, DSHB<br>Phospho-p44/42 MAPK (ERK1/2) 1:100 #4370, Cell Signaling Technology<br>anti-HA.11 Epitope Tag 1:50 901501, Biolegend<br>Chicken anti-GFP 1:800 ab13970, Abcam<br>Rabbit anti-DsRed 1:300 632496, Takara<br>anti-Chicken-AlexaFluor 488 1:300 A-11039, ThermoFisher Scientific<br>anti-Mouse-HRP 1:300 F-21453, ThermoFisher Scientific<br>anti-Rabbit-HRP 1:300 G-21234, ThermoFisher Scientific<br>anti-Fluorescein-R-phycoerythrin 1:100 A21250, Invitrogen.
Validation	All commercial antibodies were validated by the manufacturers as indicated on their websites;<br>anti-Alcama or zn-8 (https://dshb.biology.uiowa.edu/ZN-8)<br>anti-Phospho-p44/42 MAPK (ERK1/2) (https://www.cellsignal.com/products/primary-antibodies/phospho-p44-42-mapk-erk1-2-thr202-tyr204-d13-14-4e-xp-rabbit-mab/4370),<br>anti-HA. 11 Epitope Tag (https://www.biolegend.com/en-ie/products/purified-anti-ha-11-epitope-tag-antibody-11374),<br>Chicken anti-GFP (https://www.abcam.com/gfp-antibody-ab13970.html),<br>Rabbit anti-DsRed (https://www.takarabio.com/documents/Certificate%20of%20Analysis/632496/632496-101717.pdf),<br>anti-Chicken-AlexaFluor 488 (https://www.thermofisher.com/antibody/product/A-11039.html?ef_id=EAIaIQobChMI_KO8xK6I-wIVfBoGAB09Fg23EAAYASAAEgKOU_D_BwE:G:s&s_kwcid=AL!3652!3!516608152068!!!g!!!12825517856!122158234075&cid=bid_pca_aus_r01_co_cp1359_pjt0000_bid00000_0se_gaw_dy_pur_con&gclid=EAIaIQobChMI_KO8xK6I-wIVfBoGAB09Fg23EAAYASAAEgKOU_D_BwE),<br>anti-mouse-HRP (https://www.thermofisher.com/antibody/product/Goat-anti-Mouse-IgG-H-L-Secondary-Antibody-Polyclonal/F-21453),<br>anti-rabbit-HRP (https://www.thermofisher.com/antibody/product/Goat-anti-Rabbit-IgG-H-L-Cross-Adsorbed-Secondary-Antibody-Polyclonal/G-21234),<br>anti-Fluorescein-R-phycoerythrin (https://www.thermofisher.com/antibody/product/Fluorescein-Oregon-Green-Antibody-Polyclonal/A-21250)

Animals and other research organisms

Policy information about studies involving animals; ARRIVE guidelines recommended for reporting animal research, and Sex and Gender in Research

Laboratory animals	Male and female adult wild-type AB and transgenic zebrafish; Tg(ET37:EGFP), Tg(pax3a:EGFP)il50, Tg(UAS:Lifeact-GFP)mu271, Tg(7xTCF-Xla.Siam:nlsmCherry), Tg(kdrl:ras-mCherry), Tg(lmo2:DsRed), Tg(cspg4:Gal4;UAS:RFP) and Tg(ola-twist:Gal4; UAS:Kaede) were maintained at the Institut Pasteur fish facility. Reproductive age (12-18 months) of females and male zebrafish were naturally mated to obtain embryonic samples for the study. Experiments were performed with the embryos between 20 hours - 5 days of development.
Wild animals	No wild animals were used.
Reporting on sex	It is not a case for this study because the developmental stage we performed the experiment is too early to determine the sex.
Field-collected samples	No field-collected samples were used.
Ethics oversight	The experiments in this study were all carried out using embryos before 5 days of development. The general fish maintenance at the Institute follows the regulations of the 2010/63 UE European directives and is supervised by the veterinarian office of Myriam Mattei.

Note that full information on the approval of the study protocol must also be provided in the manuscript.
